# Crystal structure of magnesium copper(II) bis­[orthophosphate(V)] monohydrate

**DOI:** 10.1107/S2056989014026930

**Published:** 2015-01-01

**Authors:** Jamal Khmiyas, Abderrazzak Assani, Mohamed Saadi, Lahcen El Ammari

**Affiliations:** a Laboratoire de Chimie du Solide Appliquée, Faculté des Sciences, Université Mohammed V, Avenue Ibn Battouta, BP 1014, Rabat, Morocco; b Laboratoire de Chimie du Solide Appliquée, Faculté des Sciences, Université Mohammed V-, Avenue Ibn Battouta, BP 1014, Rabat, Morocco

**Keywords:** crystal structure, magnesium copper(II) bis­[orthophosphate(V)] monohydrate, hydrogen bonding, transition metal phosphates, hydro­thermal synthesis

## Abstract

Single crystals of magnesium copper(II) bis­[orthophosphate(V)] monohydrate, Mg_1.65_Cu_1.35_(PO_4_)_2_·H_2_O, were grown under hydro­thermal conditions. The crystal structure is formed by three types of cationic sites and by two unique (PO_4_)^3−^ anions. One site is occupied by Cu^2+^, the second site by Mg^2+^and the third site by a mixture of the two cations with an Mg^2+^:Cu^2+^ occupancy ratio of 0.657 (3):0.343 (3). The structure is built up from more or less distorted [MgO_6_] and [(Mg/Cu)O_5_(H_2_O)] octa­hedra, [CuO_5_] square-pyramids and regular PO_4_ tetra­hedra, leading to a framework structure. Within this framework, two types of layers parallel to (-101) can be distinguished. The first layer is formed by [Cu_2_O_8_] dimers linked to PO_4_ tetra­hedra *via* common edges. The second, more corrugated layer results from the linkage between [(Cu/Mg)_2_O_8_(H_2_O)_2_] dimers and [MgO_6_] octa­hedra by common edges. The PO_4_ units link the two types of layers, leaving space for channels parallel [101], into which the H atoms of the water mol­ecules protrude. The latter are involved in O—H⋯O hydrogen-bonding inter­actions (one bifurcated) with framework O atoms across the channels.

## Abstract 2

The crystal structure of magnesium copper(II) bis­[orthophosphate(V)] monohydrate is formed by three types of cationic sites and by two unique (PO_4_)^3−^ anions. One site is occupied by Cu^2+^, the second site by Mg^2+^and the third site by a mixture of the two cations with an Mg^2+^:Cu^2+^ occupancy ratio of 0.657 (3):0.343 (3).

## Chemical context   

Transition metal phosphates are an important class of mat­erials characterized by a great structural diversity originating from the presence of different coordination polyhedra *M*O_*n*_ (with *n* = 4, 5 and 6) or the possibility of phosphate groups to condense. The alternation of PO_4_ tetra­hedra and *M*O_*n*_ polyhedra can give rise to different anionic frameworks [*M* ^II^PO_4_]^−^ with pores or channels offering suitable environments to accommodate different other cations (Gao & Gao, 2005 ▸; Viter & Nagornyi, 2009 ▸). In previous studies, our focus of research was dedicated to the examination of mixed divalent orthophosphates with general formula (*M*,*M*′)_3_(PO_4_)_2_·*n*H_2_O. For instance, we have succeeded in the preparation and structure determination of some new phosphates such as Ni_2_Sr(PO_4_)_2_·2H_2_O (Assani *et al.*, 2010*a* ▸).

In the context of our main research, we report here the hydro­thermal synthesis and structural characterization of the mixed-metal orthophosphate Mg_1.65_Cu_1.35_(PO_4_)_2_·H_2_O, isolated during investigation of the qu­inter­nary system Ag_2_O/MgO/CuO/P_2_O_5_/H_2_O. The title compound crystallizes in the Fe_3_(PO_4_)_2_·H_2_O structure type (Moore & Araki, 1975 ▸) and is isotypic with other phases of the type (*M,M*’)_3_(PO_4_)_2_·H_2_O (Liao *et al.*, 1995 ▸), *viz.* Co_2.59_Zn_0.41_(PO_4_)_2_·H_2_O (Sørensen *et al.*, 2005 ▸), Co_2.39_Cu_0.61_(PO_4_)_2_·H_2_O (Assani *et al.*, 2010*b* ▸), (Cu_1−*x*_Co_*x*_)_3_(PO_4_)_2_·H_2_O (0 < *x* < 0.20 and 0.55 < *x* < 0.65), and (Cu_1−*x*_Zn_*x*_)_3_(PO_4_)_2_·H_2_O (0 < *x* < 0.19) (Viter & Nagornyi, 2006 ▸).

## Structural commentary   

**Figure 1:**
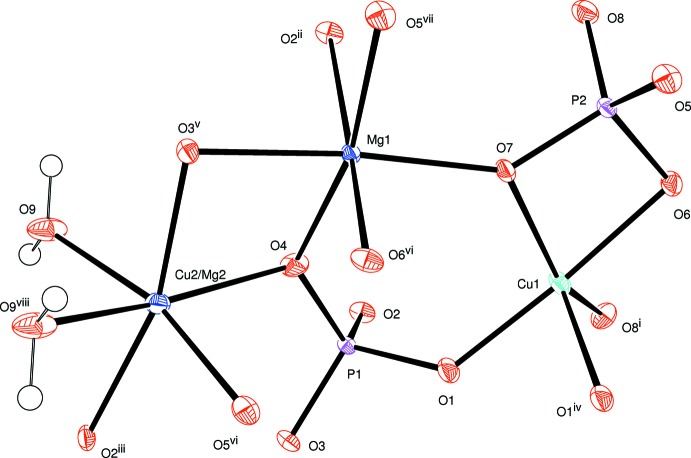
The principal building units in the crystal structure of the title compound. Displacement ellipsoids are drawn at the 50% probability level. [Symmetry codes: *x* − 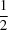, −*y* + 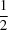, *z* − 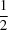; (ii) −*x* + 1, −*y* + 1, −*z* + 1; (iii) −*x* + 2, −*y* + 1, −*z* + 1; (iv) *x* + 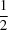, −*y* + 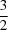, *z* + 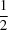; (v) −*x* + 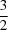, *y* + 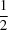, −*z* + 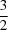; (vi) −*x* + 2, −*y* + 1, −*z* + 2; (vii) −*x* + 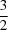, *y* + 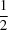, −*z* + 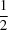; (viii) −*x* + 2, −*y* + 2, −*z* + 1.]

**Figure 2:**
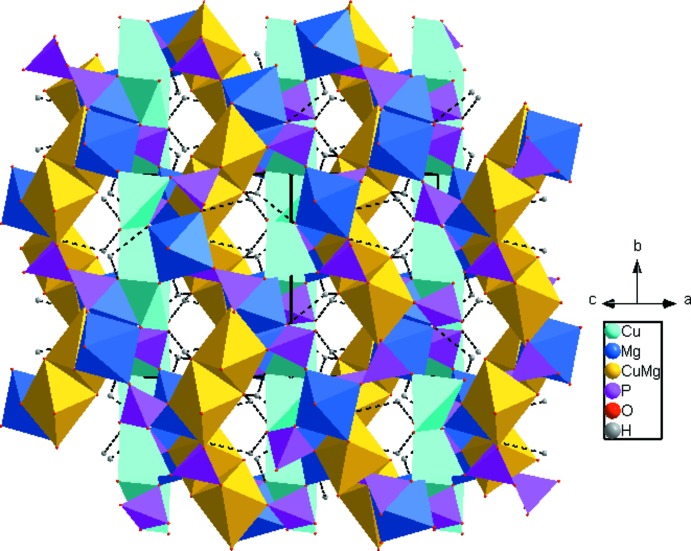
A polyhedral view of the title compound, showing the three-dimensional framework structure and O—H⋯O hydrogen bonding (dashed lines) in the channels.

The principal building units of the crystal structure of the title compound are represented in Fig. 1 ▸. The metal cations are located in three crystallographically independent sites, one octa­hedrally surrounded site entirely occupied by Mg^2+^, one site with a square-pyramidal coordination completely occupied by Cu^2+^ and one mixed-occupied (Mg^2+^/Cu^2+^) site with an octa­hedral coordination. The [Cu1O_5_] square pyramid is distorted, with Cu—O bond lengths ranging from 1.9073 (17) to 2.2782 (16) Å. Two [Cu1O_5_] polyhedra are linked together by edge-sharing to build up a [Cu_2_O_8_] dimer. By sharing corners with PO_4_ tetra­hedra, a layered arrangement parallel to (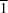
01) is formed (Fig. 2 ▸). The mixed-occupied [(Mg/Cu)O_5_(H_2_O)] octa­hedron is likewise distorted, with (Mg/Cu)—O distances varying between 2.0038 (18) and 2.384 (2) Å. Two [(Mg/Cu)O_5_(H_2_O)] octa­hedra share a common edge to built up another dimer [(Mg/Cu)_2_O_8_(H_2_O)_2_] that links [MgO_6_] octa­hedra and PO_4_ tetra­hedra *via* common vertices to build the second type of layer lying parallel to the first (Fig. 2 ▸). Adjacent layers are connected into a three-dimensional framework by common edges and vertices, and delimit channels parallel to [101], into which the hydrogen atoms of the water mol­ecules protrude. O—H⋯O hydrogen-bonding inter­actions between the water mol­ecules and framework O atoms are present (Table 1 ▸, Fig. 2 ▸).

## Synthesis and crystallization   

The title compound, Mg_1.65_Cu_1.35_(PO_4_)_2_·H_2_O, was synthesized hydro­thermally form a reaction mixture of AgNO_3_, MgO, metallic copper, and 85wt% phospho­ric acid in the molar ratio Ag: Mg: Cu: P = 1: 4: 4.5: 6 in 12.5 ml of water. The hydro­thermal reaction was conducted in a 23 ml Teflon-lined autoclave under autogenous pressure at 493 K for three days. The resulting product was filtered off, washed with deionized water and dried in air. The obtained blue crystals correspond to the title compound.

## Refinement   

Crystal data, data collection and structure refinement details are summarized in Table 2 ▸. The *M*2 site features mixed occupation by Mg^2+^ and Cu^2+^ whereas the other two cationic sites do not show any significant disorder. Refinement of the occupancy of *M*2 resulted in a ratio of Mg^2+^:Cu^2+^ = 0.657 (3):0.343 (3). The O-bound H atoms were initially located in a difference map and refined with O—H distance restraints of 0.83 (5). In the last refinement cycle, the distances were fixed at 0.86 Å and the H atoms refined in the riding-model approximation with *U*_iso_(H) set to 1.5*U*_eq_(O). The highest remaining positive and negative electron densities observed in the final Fourier map are at 0.81 Å and 0.43 Å, respectively, from Cu1.

## Supplementary Material

Crystal structure: contains datablock(s) I. DOI: 10.1107/S2056989014026930/wm5097sup1.cif

Structure factors: contains datablock(s) I. DOI: 10.1107/S2056989014026930/wm5097Isup2.hkl

CCDC reference: 1038224

Additional supporting information:  crystallographic information; 3D view; checkCIF report

## Figures and Tables

**Table 1:** Hydrogen-bond geometry (, )

*D*H*A*	*D*H	H*A*	*D* *A*	*D*H*A*
O9H9*A*O1^i^	0.86	2.22	2.867(2)	132
O9H9*A*O6^ii^	0.86	2.38	2.934(2)	123
O9H9*B*O8^iii^	0.86	1.93	2.778(2)	170

Symmetry codes: (i) 
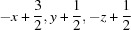
; (ii) 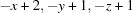; (iii) 
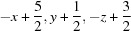
.

**Table 2:** Experimental details

Crystal data	
Chemical formula	Mg_1.65_Cu_1.35_(PO_4_)_2_H_2_O
*M*_r_	333.65
Crystal system, space group	Monoclinic, *P*2_1_/*n*
Temperature (K)	296
*a*, *b*, *c* ()	8.0701(1), 9.8661(2), 8.9944(2)
()	115.242(1)
*V* (^3^)	647.76(2)
*Z*	4
Radiation type	Mo *K*
(mm^1^)	5.16
Crystal size (mm)	0.31 0.27 0.18

Data collection	
Diffractometer	Bruker X8 APEX
Absorption correction	Multi-scan (*SADABS*; Bruker, 2009 ▸)
*T*_min_, *T*_max_	0.574, 0.748
No. of measured, independent and observed [*I* > 2(*I*)] reflections	9233, 1673, 1617
*R*_int_	0.025
(sin /)_max_ (^1^)	0.676

Refinement	
*R*[*F* ^2^ > 2(*F* ^2^)], *wR*(*F* ^2^), *S*	0.020, 0.058, 1.24
No. of reflections	1673
No. of parameters	129
H-atom treatment	H-atom parameters constrained
_max_, _min_ (e ^3^)	0.55, 0.34

Computer programs: *APEX2* and *SAINT* (Bruker, 2009 ▸), *SHELXS97* and *SHELXL97* (Sheldrick, 2008 ▸), *ORTEP-3 for Windows* (Farrugia, 2012 ▸), *DIAMOND* (Brandenburg, 2006 ▸) and *publCIF* (Westrip, 2010 ▸).

## supplementary crystallographic information

### Crystal data

**Table:**

Mg_1.65_Cu_1.35_(PO_4_)_2_·H_2_O	*F*(000) = 651
*M**_r_* = 333.65	*D*_x_ = 3.421 Mg m^−^^3^
Monoclinic, *P*2_1_/*n*	Mo *K*α radiation, λ = 0.71073 Å
Hall symbol: -p 2yn	Cell parameters from 1673 reflections
*a* = 8.0701 (1) Å	θ = 2.9–28.7°
*b* = 9.8661 (2) Å	µ = 5.16 mm^−^^1^
*c* = 8.9944 (2) Å	*T* = 296 K
β = 115.242 (1)°	Prism, blue
*V* = 647.76 (2) Å^3^	0.31 × 0.27 × 0.18 mm
*Z* = 4	

### Data collection

**Table:**

Bruker X8 APEX diffractometer	1673 independent reflections
Radiation source: fine-focus sealed tube	1617 reflections with *I* > 2σ(*I*)
Graphite monochromator	*R*_int_ = 0.025
φ and ω scans	θ_max_ = 28.7°, θ_min_ = 2.9°
Absorption correction: multi-scan (*SADABS*; Bruker, 2009)	*h* = −10→10
*T*_min_ = 0.574, *T*_max_ = 0.748	*k* = −11→13
9233 measured reflections	*l* = −12→12

### Refinement

**Table:**

Refinement on *F*^2^	Secondary atom site location: difference Fourier map
Least-squares matrix: full	Hydrogen site location: inferred from neighbouring sites
*R*[*F*^2^ > 2σ(*F*^2^)] = 0.020	H-atom parameters constrained
*wR*(*F*^2^) = 0.058	*w* = 1/[σ^2^(*F*_o_^2^) + (0.0209*P*)^2^ + 1.2623*P*] where *P* = (*F*_o_^2^ + 2*F*_c_^2^)/3
*S* = 1.24	(Δ/σ)_max_ = 0.001
1673 reflections	Δρ_max_ = 0.55 e Å^−^^3^
129 parameters	Δρ_min_ = −0.34 e Å^−^^3^
0 restraints	Extinction correction: *SHELXL97* (Sheldrick, 2008), Fc^*^=kFc[1+0.001xFc^2^λ^3^/sin(2θ)]^-1/4^
Primary atom site location: structure-invariant direct methods	Extinction coefficient: 0.0022 (6)

### Special details

**Table:**

Geometry. All e.s.d.'s (except the e.s.d. in the dihedral angle between two l.s. planes) are estimated using the full covariance matrix. The cell e.s.d.'s are taken into account individually in the estimation of e.s.d.'s in distances, angles and torsion angles; correlations between e.s.d.'s in cell parameters are only used when they are defined by crystal symmetry. An approximate (isotropic) treatment of cell e.s.d.'s is used for estimating e.s.d.'s involving l.s. planes.
Refinement. Refinement of *F*^2^ against all reflections. The weighted *R*-factor *wR* and goodness of fit *S* are based on *F*^2^, conventional *R*-factors *R* are based on *F*, with *F* set to zero for negative *F*^2^. The threshold expression of *F*^2^ > 2σ(*F*^2^) is used only for calculating *R*-factors(gt) *etc*. and is not relevant to the choice of reflections for refinement. *R*-factors based on *F*^2^ are statistically about twice as large as those based on *F*, and *R*- factors based on all data will be even larger.

### Fractional atomic coordinates and isotropic or equivalent isotropic displacement parameters (Å^2^)

**Table:**

	*x*	*y*	*z*	*U*_iso_*/*U*_eq_	Occ. (<1)
Cu1	0.64417 (4)	0.37312 (3)	0.56030 (3)	0.00781 (10)	
Mg1	0.98357 (10)	0.62883 (7)	0.72316 (9)	0.00506 (16)	
Cu2	0.88601 (7)	0.86606 (5)	0.46685 (6)	0.00771 (19)	0.343 (3)
Mg2	0.88601 (7)	0.86606 (5)	0.46685 (6)	0.00771 (19)	0.657 (3)
P1	0.70807 (7)	0.57775 (6)	0.32947 (7)	0.00456 (13)	
P2	0.88138 (7)	0.33721 (6)	0.86197 (7)	0.00517 (13)	
O1	0.5825 (2)	0.51553 (17)	0.4023 (2)	0.0088 (3)	
O4	0.8713 (2)	0.64903 (17)	0.4655 (2)	0.0092 (3)	
O3	0.5882 (2)	0.68080 (16)	0.19945 (19)	0.0068 (3)	
O2	0.7722 (2)	0.46440 (16)	0.2486 (2)	0.0071 (3)	
O5	0.8579 (2)	0.36647 (17)	1.0187 (2)	0.0088 (3)	
O6	0.7227 (2)	0.24356 (17)	0.7487 (2)	0.0081 (3)	
O7	0.8521 (2)	0.46259 (17)	0.75021 (19)	0.0078 (3)	
O8	1.0684 (2)	0.27408 (17)	0.9037 (2)	0.0094 (3)	
O9	1.1046 (2)	0.9118 (2)	0.4255 (2)	0.0139 (4)	
H9A	1.0944	0.9060	0.3265	0.021*	
H9B	1.2106	0.8782	0.4857	0.021*	

### Atomic displacement parameters (Å^2^)

**Table:**

	*U*^11^	*U*^22^	*U*^33^	*U*^12^	*U*^13^	*U*^23^
Cu1	0.01066 (15)	0.00588 (15)	0.00473 (15)	−0.00143 (9)	0.00122 (11)	0.00069 (9)
Mg1	0.0054 (3)	0.0042 (3)	0.0049 (3)	−0.0001 (2)	0.0016 (3)	0.0003 (3)
Cu2	0.0060 (3)	0.0097 (3)	0.0068 (3)	0.00074 (16)	0.00216 (19)	0.00187 (17)
Mg2	0.0060 (3)	0.0097 (3)	0.0068 (3)	0.00074 (16)	0.00216 (19)	0.00187 (17)
P1	0.0056 (2)	0.0043 (3)	0.0034 (2)	0.00024 (18)	0.00156 (19)	−0.00019 (18)
P2	0.0064 (2)	0.0046 (2)	0.0041 (2)	−0.00022 (19)	0.00177 (19)	0.00026 (19)
O1	0.0092 (7)	0.0097 (8)	0.0091 (7)	0.0016 (6)	0.0054 (6)	0.0036 (6)
O4	0.0084 (7)	0.0097 (8)	0.0062 (7)	−0.0017 (6)	−0.0001 (6)	−0.0025 (6)
O3	0.0088 (7)	0.0052 (7)	0.0050 (7)	0.0016 (6)	0.0017 (6)	0.0011 (6)
O2	0.0084 (7)	0.0061 (7)	0.0064 (7)	0.0022 (6)	0.0027 (6)	−0.0014 (6)
O5	0.0111 (8)	0.0102 (8)	0.0053 (7)	−0.0004 (6)	0.0036 (6)	−0.0014 (6)
O6	0.0091 (7)	0.0078 (7)	0.0060 (7)	−0.0027 (6)	0.0019 (6)	0.0005 (6)
O7	0.0083 (7)	0.0056 (7)	0.0071 (7)	−0.0014 (6)	0.0011 (6)	0.0021 (6)
O8	0.0086 (7)	0.0099 (8)	0.0094 (8)	0.0025 (6)	0.0035 (6)	0.0030 (6)
O9	0.0085 (7)	0.0256 (10)	0.0073 (8)	0.0035 (7)	0.0032 (6)	0.0045 (7)

### Geometric parameters (Å, º)

**Table:**

Cu1—O1	1.9073 (17)	Cu2—O3^iv^	2.0837 (16)
Cu1—O8^i^	1.9322 (17)	Cu2—O4	2.1442 (18)
Cu1—O6	1.9980 (16)	Cu2—O9^viii^	2.384 (2)
Cu1—O7	2.0169 (16)	P1—O4	1.5340 (17)
Cu1—O1^ii^	2.2782 (16)	P1—O2	1.5392 (16)
Mg1—O7	2.0236 (18)	P1—O3	1.5401 (16)
Mg1—O2^iii^	2.0898 (17)	P1—O1	1.5491 (17)
Mg1—O4	2.1070 (18)	P2—O8	1.5241 (17)
Mg1—O3^iv^	2.1071 (17)	P2—O5	1.5279 (17)
Mg1—O6^v^	2.1156 (17)	P2—O7	1.5473 (17)
Mg1—O5^vi^	2.1205 (18)	P2—O6	1.5550 (17)
Cu2—O9	2.0038 (18)	O9—H9A	0.8600
Cu2—O5^v^	2.0268 (17)	O9—H9B	0.8600
Cu2—O2^vii^	2.0535 (17)		
			
O1—Cu1—O8^i^	96.29 (7)	O5^v^—Cu2—O2^vii^	80.18 (7)
O1—Cu1—O6	172.25 (7)	O9—Cu2—O3^iv^	82.06 (7)
O8^i^—Cu1—O6	91.43 (7)	O5^v^—Cu2—O3^iv^	107.59 (7)
O1—Cu1—O7	99.62 (7)	O2^vii^—Cu2—O3^iv^	163.32 (7)
O8^i^—Cu1—O7	146.81 (7)	O9—Cu2—O4	105.96 (8)
O6—Cu1—O7	73.33 (7)	O5^v^—Cu2—O4	87.11 (7)
O1—Cu1—O1^ii^	77.52 (7)	O2^vii^—Cu2—O4	117.13 (7)
O8^i^—Cu1—O1^ii^	116.46 (6)	O3^iv^—Cu2—O4	78.56 (6)
O6—Cu1—O1^ii^	99.65 (6)	O9—Cu2—O9^viii^	89.45 (7)
O7—Cu1—O1^ii^	95.41 (6)	O5^v^—Cu2—O9^viii^	80.53 (6)
O7—Mg1—O2^iii^	98.29 (7)	O2^vii^—Cu2—O9^viii^	81.28 (6)
O7—Mg1—O4	101.96 (7)	O3^iv^—Cu2—O9^viii^	85.44 (6)
O2^iii^—Mg1—O4	96.67 (7)	O4—Cu2—O9^viii^	155.82 (7)
O7—Mg1—O3^iv^	171.09 (8)	O4—P1—O2	111.26 (9)
O2^iii^—Mg1—O3^iv^	90.39 (7)	O4—P1—O3	110.54 (9)
O4—Mg1—O3^iv^	78.89 (7)	O2—P1—O3	110.47 (9)
O7—Mg1—O6^v^	86.54 (7)	O4—P1—O1	109.79 (9)
O2^iii^—Mg1—O6^v^	166.01 (7)	O2—P1—O1	108.93 (9)
O4—Mg1—O6^v^	95.14 (7)	O3—P1—O1	105.69 (9)
O3^iv^—Mg1—O6^v^	84.55 (7)	O8—P2—O5	110.47 (9)
O7—Mg1—O5^vi^	89.35 (7)	O8—P2—O7	110.33 (9)
O2^iii^—Mg1—O5^vi^	77.24 (7)	O5—P2—O7	113.81 (9)
O4—Mg1—O5^vi^	167.90 (8)	O8—P2—O6	111.75 (10)
O3^iv^—Mg1—O5^vi^	90.60 (7)	O5—P2—O6	108.96 (9)
O6^v^—Mg1—O5^vi^	89.76 (7)	O7—P2—O6	101.22 (9)
O9—Cu2—O5^v^	165.32 (8)	H9A—O9—H9B	104.9
O9—Cu2—O2^vii^	87.74 (7)		

Symmetry codes: (i) *x*−1/2, −*y*+1/2, *z*−1/2; (ii) −*x*+1, −*y*+1, −*z*+1; (iii) −*x*+2, −*y*+1, −*z*+1; (iv) *x*+1/2, −*y*+3/2, *z*+1/2; (v) −*x*+3/2, *y*+1/2, −*z*+3/2; (vi) −*x*+2, −*y*+1, −*z*+2; (vii) −*x*+3/2, *y*+1/2, −*z*+1/2; (viii) −*x*+2, −*y*+2, −*z*+1.

### Hydrogen-bond geometry (Å, º)

**Table:**

*D*—H···*A*	*D*—H	H···*A*	*D*···*A*	*D*—H···*A*
O9—H9*A*···O1^vii^	0.86	2.22	2.867 (2)	132
O9—H9*A*···O6^iii^	0.86	2.38	2.934 (2)	123
O9—H9*B*···O8^ix^	0.86	1.93	2.778 (2)	170

Symmetry codes: (iii) −*x*+2, −*y*+1, −*z*+1; (vii) −*x*+3/2, *y*+1/2, −*z*+1/2; (ix) −*x*+5/2, *y*+1/2, −*z*+3/2.
